# 102. Optimizing the Sensitivity of Detection of Respiratory Syncytial Virus Infections in Longitudinal Studies Using the Combination of Weekly Sample Testing and Biannual Serology

**DOI:** 10.1093/ofid/ofaf695.037

**Published:** 2026-01-11

**Authors:** Shannon C Conrey, Daniel C Payne, Maria P Deza Leon, Monica Epperson, Coughlin Melissa, Burrell R Allison, Claire Mattison, Julia M Baker, Natalie J Thornburg, Meredith L McMorrow, Mary A Staat, Ardythe L Morrow

**Affiliations:** Case Western Reserve University, Cleveland, Ohio; Cincinnati Children's Hospital Medical Center, Decatur, GA 30030-3637, Georgia; Children's Mercy Hospital, Kansas City, Missouri; Centers for Disease Control and Prevention, Atlanta, Georgia; CDC, Atlanta, GA, Georgia; Cincinnati Children's Medical Center Hospital, Cincinnati, Ohio; Centers for Disease Control, Atlanta, Georgia; Centers for Disease Control and Prevention, Atlanta, Georgia; Centers for Disease Control and Prevention, Atlanta, Georgia; CDC/NCIRD/CORVD/SPB, Atlanta, GA; Cincinnati Children's Hospital Medical Center, Decatur, GA 30030-3637, Georgia; University of Cincinnati College of Medicine, Cincinnati, Ohio

## Abstract

**Background:**

Cohort studies with frequent sampling and testing can improve the full capture of infections and disease burden but are often challenged by incomplete adherence to sampling regimens. We describe the detection sensitivity of respiratory syncytial virus (RSV) infection achieved in a birth cohort using a combination of weekly nasal sample testing and serology.

**Figure:**
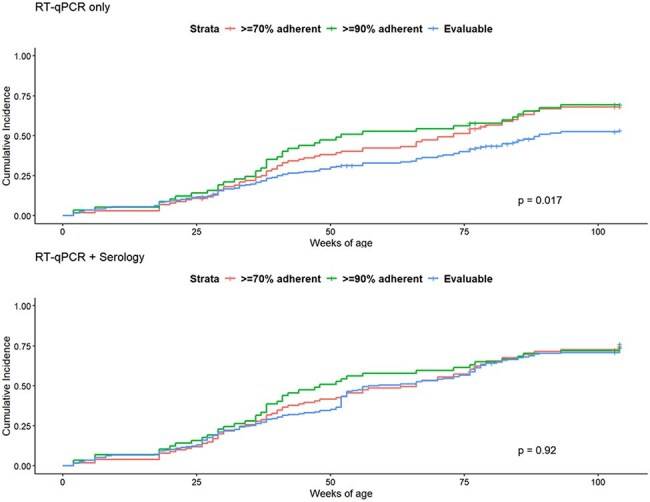
Comparison of Cumulative Incidence by Weekly Sample Adherence Level using RT-qPCR -Only Detections and a Combination of RT-qPCR and Biannual Serology Cumulative incidence comparisons by subset adherence level demonstrating the differences among adherence levels in cumulative incidence when using RT-qPCR only to detect RSV infections (top) and the attenuation of differences when using a combination of RT-qPCR and serological RSV detections (bottom). P-values in the plots represents differences by log-likelihood. All participants participated in the study for at least 18 months Blue: participants who submitted at least 70% of weekly samples OR provided a serum sample at 18 or 24 months of age Coral: particpants who submitted at least 70% of weekly samples Green: participant who submitted at least 90% of weekly samples

**Figure:**
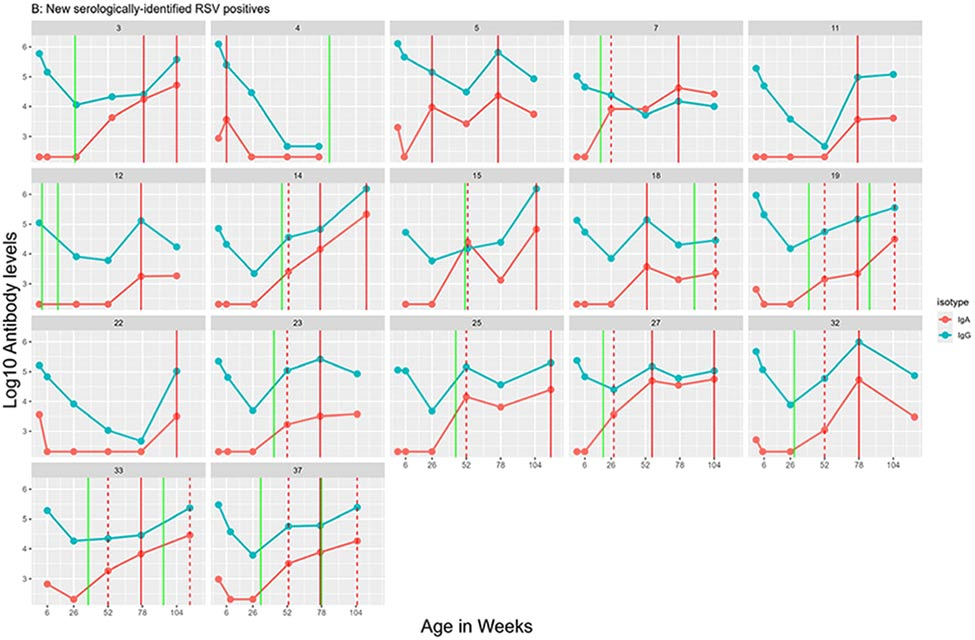
New serologically identified infections in highly adherent PREVAIL children Using CART-derived anti-RSV IgA and IgG thresholds, we identified 21 infections in 17 children that were not previously detected using real-time polymerase chain reaction (RT-qPCR) testing. These infections were detected among children who submitted at least 90% of their weekly samples for testing. Green solid line: RT-qPCR detected RSV infection, Red dotted line: serologically-confirmed RT-qPCR infection, Red solid line: new serologically-detected infection.

**Methods:**

The PREVAIL Cohort (2017-2020 Cincinnati, OH) followed mothers and their healthy babies from birth to age two. Mothers collected mid-turbinate nasal swabs weekly which were tested for RSV using real-time polymerase chain reaction (RT-qPCR). Serum was collected at age 6 weeks and biannually from 6-24 months and tested for RSV pre-fusion F IgG and IgA antibody. Mixed effects classification and regression trees (CART) identified IgG and IgA thresholds consistent with a RT-qPCR-identified RSV infection using a subset of participants having ≥ 90% weekly sample adherence. Resulting thresholds were applied to participants with ≥ 18 months of follow-up who provided either ≥ 70% of weekly samples or serum at age 18 to 24 months. Incidence rates by adherence strata and detection method were compared using Fisher’s exact test.

**Figure:**
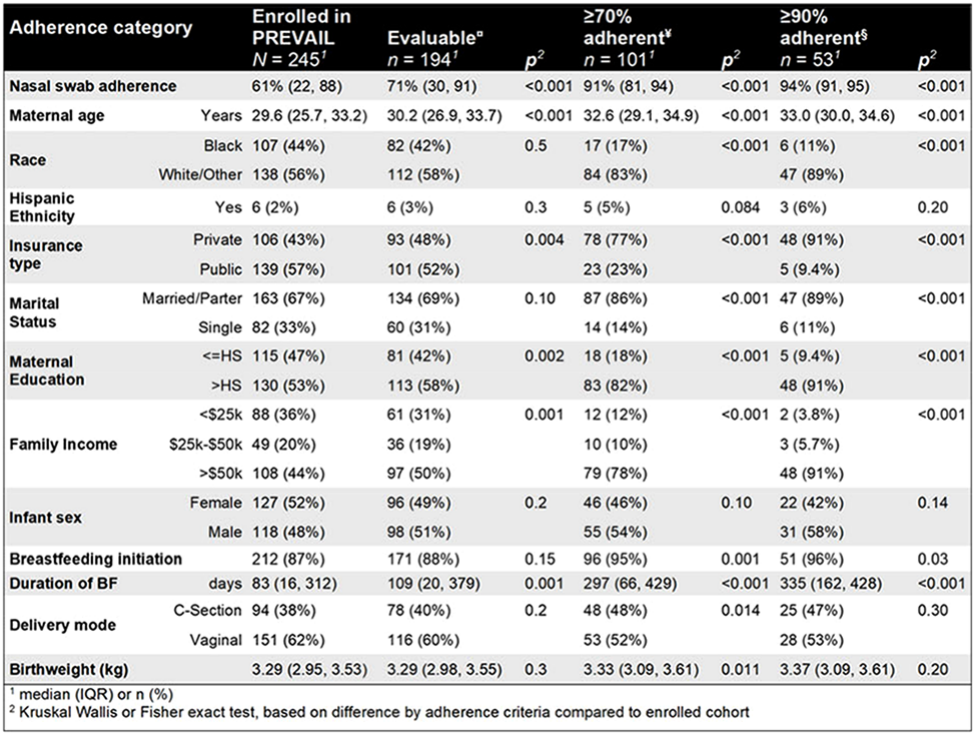
Demographics of All Enrolled and Each Weekly Sample Adherence Subset

Adherence categories were all subsets of the 245 enrolled participants, thus each subset was nested within the larger groupings. The p-values represent the difference between those who meet each adherence category and all enrolled participants.

¤Evaluable participants were under active follow-up for at least 18 months and either submitted ≥70% of weekly samples or provided a serum sample ≥18 months of age ¥≥70% adherent participants were under active follow-up for at least 18 months and submitted ≥70% of weekly samples. To minimize missed infections, this group is used for analysis using only PCR-identified infections §≥90% adherent participants were under active follow-up for at least 18 months and submitted ≥90% of weekly samples. This group is used when true positives and negatives are required Power achieved by adherence criteria

**Figure:**
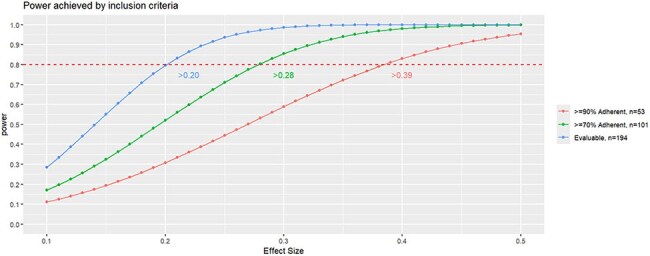

Comparison of power achieved using chi-squared tests by each of the the adherence categories. By using a combination of weekly nasal swabs tested using RT-qPCR and biannual serology, power can be maximized by including participants with lower weekly sample submission adherence.

Evaluable participants included all who participated in PREVAIL at least 18 months and provided either at least 70% of weekly samples or a serum sample at 18 or 24 months of age (n=194) >=70% adherent participants were evaluable participants who submitted at least 70% of weekly samples (n=101) >=90% adherent participants were evaluable participants who submitted at least 90% of weekly samples (n=53)

**Results:**

Of 245 enrolled participants, 194 (79%) met inclusion criteria, with 101 (41%) providing ≥ 70% of samples and 53 (22%) providing ≥ 90% of samples. CART identified a log_10_ change in IgG > 0.32 or IgA > 0.20, providing 95% sensitivity and 100% specificity for identifying RSV seropositivity. Comparing RT-qPCR-only to a combination of RT-qPCR and serology, cumulative incidence (49% vs 75%, *p* < 0.001) and incidence density increased (0.33 vs 0.71 infections/child-year, *p* < 0.001). Differences in cumulative incidence and incidence density were found when comparing adherence strata using RT-qPCR detections only, but were attenuated when combining RT-qPCR and serologic detections.

**Conclusion:**

CART-identified thresholds in serum antibody accurately identified incident RSV infections, capturing a more robust estimate of disease burden, increasing power and reducing potential bias associated with sample adherence.

**Disclosures:**

All Authors: No reported disclosures

